# Marked Improvement of Chronic Motor and Phonic Tics With Cariprazine in an Adult With Tourette Syndrome With Comorbid Bipolar Disorder: A Case Report

**DOI:** 10.7759/cureus.109023

**Published:** 2026-05-17

**Authors:** Gavin L Feinberg, George Burton, Jennifer J Hawkins, Jorge L Juncos

**Affiliations:** 1 Neurology, Juncos Neuro Consulting, Cumming, USA; 2 Biological Sciences, Augusta University, Augusta, USA; 3 Counseling, Hawkins Counseling, Cumming, USA; 4 Neurology, Emory University School of Medicine, Atlanta, USA

**Keywords:** bipolar disorder, cariprazine, chronic motor tics, chronic phonic tics, dopamine partial agonist, tic disorder, tourette syndrome

## Abstract

Tourette syndrome (TS) is a neuropsychiatric disorder characterized by motor and phonic tics, often complicated by comorbid conditions and treatment-related adverse effects. We report a 61-year-old male with long-standing TS and comorbid bipolar disorder who experienced persistent, functionally impairing tics despite multiple prior therapies. Following initiation and titration of cariprazine, the patient demonstrated marked and sustained improvement in tic severity over approximately two years, with good tolerability and stable mood symptoms. This case highlights a potential role for cariprazine in the management of tic disorders in patients with complex neuropsychiatric comorbidities.

## Introduction

Tic disorders, including Tourette syndrome (TS), are characterized by sudden, repetitive motor movements or vocalizations, often accompanied by premonitory urges and experienced by patients as difficult to suppress. Symptoms typically emerge in childhood or early adolescence and follow a fluctuating course, with waxing and waning over time. Although tic severity often diminishes with age and may stabilize in adulthood, a subset of patients continues to experience persistent and functionally impairing symptoms that require treatment [1].

Pharmacologic treatment is generally reserved for patients with moderate-to-severe or functionally impairing tics. Current guidelines emphasize individualized treatment selection, counseling about expected benefits and harms, and careful monitoring for adverse effects, including weight gain, sedation, prolactin elevation, drug-induced movement disorders, hypotension, bradycardia, and electrocardiographic changes [2,3]. Commonly used medications include dopamine receptor antagonists or partial agonists such as risperidone, aripiprazole, and pimozide; however, tolerability can limit long-term use, particularly in adults with complex psychiatric comorbidity [1-3].

TS also frequently occurs with neuropsychiatric comorbidities, most notably attention-deficit/hyperactivity disorder (ADHD) and obsessive-compulsive disorder (OCD), and many patients have mood, anxiety, or substance-use disorders that complicate clinical management. In a large TS genetics consortium study, most individuals with TS had at least one psychiatric comorbidity, and more than half had two or more, underscoring that treatment response is often shaped by both tic burden and broader psychiatric stability [4].

Cariprazine is a dopamine D3/D2 partial agonist with higher binding affinity for D3 than D2 receptors, as well as 5-HT1A partial agonist and 5-HT2A antagonist activity [5,6]. This receptor profile distinguishes it from traditional dopamine receptor antagonists and may be clinically relevant in patients who require management of both tic symptoms and mood instability. However, the relevance of this pharmacologic profile to TS remains uncertain, and receptor-level mechanisms cannot be inferred from isolated clinical observations.

Cariprazine is not currently approved for TS. Published evidence remains extremely limited, with one prior case report describing improvement in an adolescent with TS, ADHD, and depression after cariprazine was introduced because of inadequate response or tolerability problems with other agents [7]. Given this limited evidence base, we present this adult case as a hypothesis-generating report of cariprazine use in a patient with persistent, functionally impairing tics, bipolar disorder, and prior adverse effects from dopamine-blocking therapy.

## Case presentation

A 61-year-old male with childhood-onset TS, characterized by predominantly phonic and facial motor tics, presented for the management of persistent symptoms. His tics consisted of repetitive, forceful coughing, throat clearing, and sniffing, without identifiable triggers or relief following the behaviors. The symptoms were often continuous and interfered with social functioning in youth and later with professional activities. High-amplitude limb tics were not observed.

His history was notable for features consistent with ADHD and a longstanding pattern of anxiety. He had carried a diagnosis of bipolar disorder since his late 20s, characterized by intermittent episodes of mild mania, including insomnia, agitation, and increased compulsive behaviors. Periods of alcohol misuse and compulsive sexual behavior occurred several times in adulthood, often in the setting of mood destabilization or medication-related side effects. At the time of presentation, bipolar symptoms remained stable on lamotrigine, and compulsive behaviors had been controlled with mood stabilizers and psychotherapy.

Prior to neurologic evaluation, tics were managed with pimozide and high-dose lurasidone, which provided only partial symptom control and were limited by adverse effects. At higher doses of pimozide, he developed drug-induced Parkinsonian features, including bradykinesia and facial hypomimia.

At presentation, his medication regimen included pimozide 2 mg twice daily, lurasidone 160 mg/day, lamotrigine 200 mg/day, and clonazepam 3 mg nightly for insomnia and relaxation. Given persistent tic burden and concerns regarding adverse effects associated with dopamine receptor antagonists, cariprazine was initiated as an off-label adjunctive treatment.

Cariprazine was started at 1.5 mg daily and titrated to 3 mg daily over two weeks, while lurasidone was gradually reduced from 160 mg/day to 120 mg/day. Pimozide and clonazepam were continued during this early period. Within three to five weeks, the patient reported a reduction in tic frequency and intensity, with previously continuous symptoms becoming intermittent and less forceful.

Cariprazine was subsequently increased to 4.5 mg/day, with a plan to increase to 6 mg/day. By approximately three months, lurasidone had been reduced to 80 mg/day, and reductions in pimozide and clonazepam were underway. Tics were described as mild, with persistent but less disruptive phonic symptoms, and mood symptoms remained stable.

By approximately five months, cariprazine had been increased to 6 mg/day. During this period, lurasidone had been reduced and then resumed after the recurrence of compulsive behaviors. Pimozide was reduced over several months but was not fully stopped. Tic control remained improved compared with baseline, although the clinical course was complicated by medication adjustments affecting both tic and psychiatric symptoms.

At approximately two-year follow-up, the patient remained clinically improved compared with baseline. His regimen included cariprazine 4.5 mg daily, pimozide 2 mg twice daily, lurasidone 120 mg daily, and clonazepam 4 mg nightly. Tics remained mild and stable relative to baseline, and bipolar symptoms were controlled after restoration of lurasidone and pimozide.

Physical examination revealed eventual improvement of tremor and rigidity after approximately six months. This improvement was interpreted in the context of reduced dopamine-blocking medication burden and longitudinal regimen optimization rather than as a direct therapeutic effect of cariprazine. Mild facial hypomimia and probable medication-related imbalance persisted, with no reported akathisia or dyskinesia (Table 1).

**Table 1:** Clinical course, medication adjustments, and estimated tic severity. * Scores were not formally administered but retrospectively estimated from clinical chart review to demonstrate general severity trends. They are intended to approximate longitudinal change rather than represent prospectively collected Yale Global Tic Severity Scale (YGTSS) measurements [8].

Time point	Medication regimen	Estimated YGTSS score* (0-50)	Clinical tic severity	Clinical findings
Baseline	Pimozide 2 mg twice daily; lurasidone 160 mg/day; lamotrigine 200 mg/day; clonazepam 3 mg nightly	40-45	Severe	Continuous, loud phonic tics, including coughing and sniffing, with facial grimacing; prior drug-induced Parkinsonism
Weeks 2-4	Cariprazine 1.5→3 mg/day; lurasidone reduced from 160 to 120 mg/day; pimozide continued; clonazepam continued	25-30	Moderate	Reduced tic frequency and intensity following initiation of cariprazine
Month 3	Cariprazine 4.5 mg/day with plan to increase to 6 mg/day; lurasidone reduced to 80 mg/day; pimozide and clonazepam reduction underway	15-20	Mild	Persistent but less disruptive phonic tics; stable mood symptoms
Month 5	Cariprazine 6 mg/day; lurasidone had been reduced and then resumed after recurrence of compulsive behaviors; pimozide was reduced over several months but not fully stopped	10-15	Mild	Sustained tic control; emergence of compulsive behaviors with medication adjustments
Approximately 2-year follow-up	Cariprazine 4.5 mg/day; pimozide 2 mg twice daily; lurasidone 120 mg/day; clonazepam 4 mg nightly	10-15	Mild, sustained improvement	Tics remained improved and clinically stable compared with baseline. Bipolar symptoms were controlled after restoration of lurasidone and pimozide.

## Discussion

This case describes an adult with long-standing TS, bipolar disorder, compulsive behaviors, anxiety, and prior medication-related Parkinsonian features who experienced sustained improvement in chronic motor and predominantly phonic tics after cariprazine was added to a complex dopamine-modulating regimen. The observation is clinically notable because the patient had persistent, functionally impairing symptoms despite treatment with guideline-supported dopamine receptor antagonists. However, improvement occurred in the setting of concurrent medication adjustments, including changes in lurasidone, pimozide, and clonazepam. The clinical course should therefore be interpreted as a hypothesis-generating association rather than evidence of cariprazine efficacy as an isolated intervention.

This observation should be considered in the context of current TS treatment literature. Pharmacologic treatment is generally reserved for patients with moderate-to-severe or functionally impairing tics, and commonly used agents include dopamine receptor antagonists or partial agonists such as risperidone, aripiprazole, and pimozide [1-3]. These medications can be effective, but treatment selection is often limited by adverse effects such as sedation, weight gain, metabolic effects, akathisia, extrapyramidal symptoms, prolactin elevation, hypotension, and electrocardiographic concerns [1-3]. In this patient, continued tic burden, prior Parkinsonian features during dopamine-blocking therapy, and the need to maintain psychiatric stability complicated standard treatment selection. Cariprazine was therefore considered as an off-label adjunctive option in a patient for whom conventional dopamine-blocking treatment had provided incomplete benefit and tolerability concerns. This rationale does not imply that cariprazine should precede established therapies, but rather explains why it was clinically considered in this unusually complex case.

The closest published comparison is the case report by Gill, which described improvement with cariprazine in an adolescent with TS, ADHD, and depression after difficulty with prior therapies [7]. The present case differs because the patient was an older adult with decades of chronic symptoms, comorbid bipolar disorder and compulsive behaviors, prior drug-induced Parkinsonian features, and approximately two years of follow-up after cariprazine initiation. Taken together, these cases do not establish efficacy or define an evidence-based treatment pathway. Instead, they suggest that cariprazine may warrant further systematic study in select TS patients with overlapping tic, mood, and behavioral symptoms, particularly when conventional dopamine-blocking treatment has been limited by adverse effects or incomplete response [7].

Cariprazine’s receptor profile provides a theoretical reason for interest in tic disorders, but the mechanistic interpretation from this case should remain limited. Cariprazine is a dopamine D3/D2 partial agonist with additional 5-HT1A partial agonist and 5-HT2A antagonist activity [5,6]. Dopaminergic dysfunction has long been implicated in TS, particularly in relation to cortico-striatal circuits involved in tic expression and behavioral regulation [9]. However, this single uncontrolled case cannot determine whether D3 receptor activity, broader dopaminergic modulation, psychiatric stabilization, or medication optimization contributed most to the observed improvement. The clinical value of the case is therefore primarily observational: tic improvement was sustained relative to baseline in a patient who required ongoing management of both tic symptoms and psychiatric comorbidity.

This case also raises a practical tolerability point. The patient previously developed Parkinsonian features during dopamine-blocking treatment. Improvement in tremor and rigidity after approximately six months was most plausibly related to reduced dopamine-blocking medication burden and longitudinal regimen optimization rather than a direct therapeutic effect of cariprazine. The more relevant observation is that cariprazine was tolerated without reported akathisia, dyskinesia, or recurrent clinically significant Parkinsonism while tic control remained improved relative to baseline. This observation is clinically useful but should not be generalized without caution, as cariprazine can cause extrapyramidal symptoms and akathisia, and its active metabolites have long half-lives that require gradual titration and careful monitoring [5].

Several limitations should be acknowledged. This is a single case report, and the Yale Global Tic Severity Scale (YGTSS) scores were retrospectively estimated from clinical chart review rather than formally administered at each time point [8]. The patient was also receiving multiple concurrent medications, including lurasidone, pimozide, lamotrigine, and clonazepam, with medication adjustments occurring during the same period as cariprazine titration. Improvement may therefore reflect combined pharmacologic effects, reduction of poorly tolerated agents, natural waxing and waning of TS, regression to the mean, or changes in comorbid psychiatric symptoms. The recurrence of compulsive behaviors during medication adjustment and subsequent stabilization after restoration of lurasidone and pimozide further emphasizes that the observed course reflects complex medication optimization rather than a clean single-agent response.

Overall, this case contributes to the current literature as a hypothesis-generating report rather than evidence of established efficacy. Its significance is not that cariprazine should replace guideline-supported treatments, but that sustained improvement was observed after cariprazine was introduced in a complex adult patient with persistent tics, psychiatric comorbidity, prior medication-related Parkinsonian features, and ongoing need for multidrug psychiatric management. Further systematic study would be required to determine whether cariprazine has a reproducible role in tic disorders and to clarify which patient subgroups, if any, may benefit [1-7,9].

## Conclusions

Cariprazine was associated with sustained improvement in chronic motor and phonic tics in an adult patient with TS, bipolar disorder, compulsive behaviors, and prior medication-related Parkinsonian features. At approximately two-year follow-up, tic severity remained improved relative to baseline, although the patient continued to require a multidrug regimen including pimozide, lurasidone, and clonazepam for tic and psychiatric stability.

This case should be interpreted cautiously because of its uncontrolled single-patient design, retrospectively estimated YGTSS scores, natural waxing and waning of TS, and multiple concurrent medication adjustments. The findings do not establish cariprazine efficacy or a specific D3-mediated mechanism. Rather, they provide a hypothesis-generating observation supporting further systematic evaluation of cariprazine in carefully selected patients with persistent tics, psychiatric comorbidity, and tolerability limitations with standard dopamine-blocking therapies.
